# Surfactant-assisted solvothermal synthesis of pure nickel submicron spheres with microwave-absorbing properties

**DOI:** 10.1186/s11671-016-1562-y

**Published:** 2016-07-29

**Authors:** Heng Guo, Bingxue Pu, Haiyuan Chen, Jin Yang, Yajun Zhou, Jian Yang, Boateng Bismark, Handong Li, Xiaobin Niu

**Affiliations:** State Key Laboratory of Electronic Thin Film and Integrated Devices, University of Electronic Science and Technology of China, Chengdu, 610054 China

**Keywords:** Solvothermal method, Nickel nanostructures, Microwave absorption properties

## Abstract

Pure metallic nickel submicron spheres (Ni-SSs), flower-like nickel nanoflakes, and hollow micrometer-sized nickel spheres/tubes were controllably synthesized by a facile and efficient one-step solvothermal method with no reducing agent. The characteristics of these nickel nanostructures include morphology, structure, and purification. Possible synthesis mechanisms were discussed in detail. The resultant Ni-SSs had a wide diameter distribution of 200~800 nm through the aggregation of small nickel nanocrystals. The ferromagnetic behaviors of Ni-SSs investigated at room temperature showed high coercivity values. Furthermore, the microwave absorption properties of magnetic Ni-SSs were studied in the frequency range of 0.5–18.0 GHz. The minimum reflection loss reached −17.9 dB at 17.8 GHz with a thin absorption thickness of 1.2 mm, suggesting that the submicron spherical structures could exhibit excellent microwave absorption properties. More importantly, this one-pot synthesize route provides a universal and convenient way for preparation of larger scale pure Ni-SSs, showing excellent microwave absorption properties.

## Background

With the rapid development of nanoscience and nanotechnology, magnetic nanostructured materials have attracted significant interest because of their interesting optical, electrical, and catalytic properties, as well as response and manipulability under magnetic fields [1–3]. As a result of these characteristics, their potential applications have been proposed in several fields such as optoelectronics, magnetics, catalysis, biologic engineering, information storage, and photovoltaic technology [4–6]. In particular, extensive research has been focused on magnetic metallic nanoparticles such as nickel (Ni), cobalt, and iron which represent a group of promising nanomaterials to ascertain their usefulness in several areas such as high density magnetic recording media, chemical and photochemical catalysis, ferrofluids, and medical diagnostics because of their special structures and distinctive magnetic and physical properties [7–9].

As an important class of ferromagnetic transition metal, Ni nanoparticles are emerging and displaying many characteristics such as high magnetism, high surface area, large surface energy, excellent chemical stability, low melting point, resource-rich, and low cost [10–12]. They are widely used in several important technological fields such as magnetic materials catalysts, magnetic fluids, microwave devices, and high-sensitive gas sensors [13–15]. Specifically, metal Ni nanoparticles as an important conducting and magnetic-anisotropy material have attracted wide significant interest because of their intriguing electronic and magnetic properties under magnetic fields [16, 17]. Over the past several years, Ni nanomaterials provide the possibility of a good candidate for microwave absorbers due to their proper combination of dielectric and magnetic loss leading to wave impedance matching. It is commonly known that the microwave absorption properties of the electromagnetic wave absorbers depend on the complex permittivity and permeability [18]. Nevertheless, it is relatively difficult to achieve impedance match conditions under many conditions owing to unilateral dielectric loss or magnetic loss [19]. For example, some studies have illustrated that an array of Ni nanowires has a negative magnetic permeability as well as a negative permittivity in the resonance frequency [20]. In fact, the attenuated permeability and weak magnetocrystalline anisotropy may limit its applications at higher frequencies due to the dimension decrease and the repression of skin effect [21]. Furthermore, this phenomenon obviously shows that the physical and chemical performances of Ni nanomaterials strongly depend on the makeup, structure, size, nanoscale morphology, and polydispersity [22, 23] which are the key factors for its further application including the electromagnetic wave absorption characteristic and intensity. However, the control of the Ni/Ni-based nanomaterials fabrication is sensitive to the preparation methods.

In recent years, multiple methods are adopted for the fabrication of Ni nanomaterials, which include sputtering, solution glow discharge process, thermal decomposition, pulsed laser ablation, and metal evaporation condensation [24–26]. To date, Ni nanomaterials have been synthesized by using different controllable synthesis routes for various applications with different purities, sizes, structures, and shapes, such as nanowires, nanoparticles, nanotubes, nanosheets, hollow microspheres, and microstructures [27–29]. Although the abovementioned Ni-based nanomaterials have been made, to our knowledge, there are only few reports on the synthesis of pure Ni nanoparticles. However, the successful routes for fabrication of the desired uniform architectures are usually complicated, which always involve the use of various surfactants and still give unstable assemblies with the small nanocrystal [30]. In addition, the metallic Ni nanoparticles are easily oxidized in the presence of nonmagnetic components [31]. In spite of their high electrocatalytic activity, these nanomaterials often have certain disadvantages including poor stability and high cost [32]. Herein, many efforts have been directed toward synthesizing metal Ni nanoparticles with a rapid, simple, economical, well-reproducible, and green way.

In this work, the flower-like Ni nanoflakes, hollow micrometer-sized Ni spheres/tubes, and Ni submicron spheres (Ni-SSs) were synthesized by a facile and efficient one-step solvothermal method with no reducing agent and only one surfactant used. The structure and morphology of the series of resultant Ni nanostructures were characterized. The possible formation mechanism for three Ni hierarchical architectures was proposed, which was confirmed by the controllable surfactant-assisted synthesis routes in addition of poly (ethylene glycol) (PEG) agents. Importantly, the pure Ni-SSs were obtained successfully. Furthermore, the microwave properties of the as-made pure Ni-SSs were investigated in a wide frequency range, and it was found that the pure Ni-SSs exhibit a strong and broad electromagnetic absorption peak with excellent microwave properties. More importantly, our findings inspire a universal and convenient way, which not only controls the low-cost reaction process of pure Ni metal-based nanomaterials as microwave absorbers but also presents operational simplicity for the large-scale production.

## Methods

### Materials

Nickel chloride hexahydrate (NiCl_2_·6H_2_O), ethylene glycol (HOCH_2_CH_2_OH, EG), anhydrous sodium acetate (CH_3_COONa, NaAc), and PEG (*M*_n_ = 600, 2000, 6000, 10,000) were purchased from Kelong Regent Co. Ltd, Chengdu, China. All the chemicals were used without any further purification.

### Synthesis of Nickel Submicron Spheres

**Fig. 1:**
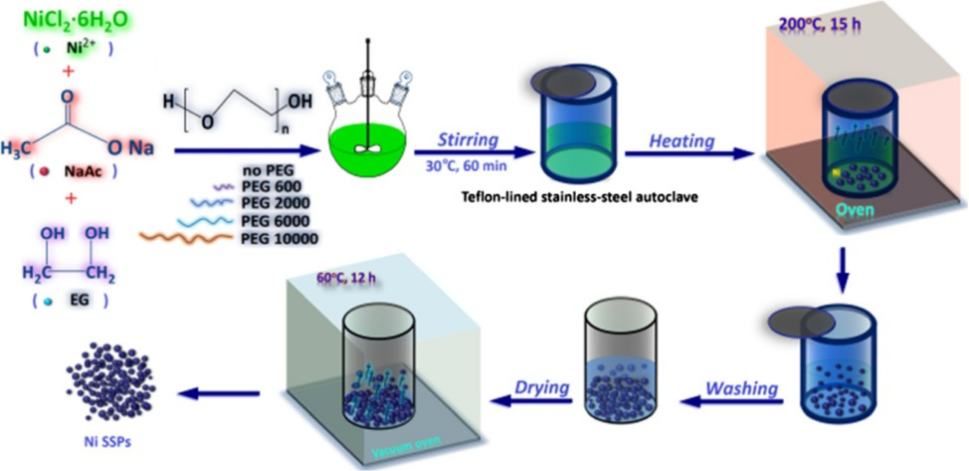
Schematic illustration of the fabrication process of the Ni submicron spheres

Nickel submicron spheres (Ni-SSs) were fabricated by a one-step process through a solvent-thermal route. As schematically illustrated in Fig. 1, a typical procedure is described as follows: NiCl_2_·6H_2_O (5.94 g) was dissolved in 160 ml of EG in a 500-ml three-neck flask at room temperature, followed by the addition of NaAc (18.0 g). The mixture was stirred vigorously for 30 min at 30 °C to form a light green solution. The solution was obtained without the addition of any other PEG (no PEG). Then, the PEG 600, PEG 2000, PEG 6000, and PEG 10,000 with the same molar (0.0025 mol) were introduced into the abovementioned solution followed by another intensive stirring of 60 min at 60 °C. Afterwards, series of the as-made solutions were sealed in a Teflon-lined stainless-steel autoclave. The autoclave was heated and maintained at 200 °C for 15 h, then cooled down to room temperature naturally. The resultant products (labelled as Ni-0, Ni-600, Ni-2000, Ni-6000, and Ni-10000, respectively) were collected by centrifugation, rinsed several times with absolute ethanol and distilled water, separately, and dried in a vacuum oven at 60 °C for 12 h.

### Characterization

The morphologies of the obtained samples were observed by scanning electron microscopy (SEM) (JSM, 6490LV). An energy dispersive X-ray spectrometer (EDS, EDAX GENESIS 2000XMS) associated with SEM was used to characterize the compositions of the samples. Transmission electron microscopy (TEM) and high-resolution TEM (HRTEM) images were acquired using a JEOL JEM 2010F microscope working at 200 kV. The obtained samples were further characterized by X-ray powder diffractometer (XRD) (Rigaku RINT2400 with CuKα radiation). Thermal gravimetric analysis (TGA) was performed on a TA Instruments Q50. The samples (10–15 mg) were heated from ambient temperature to 500 °C at a rate of 20 °C·min^−1^ under nitrogen flow with a purge of 40 ml min^-1^. The magnetic properties were examined using a vibrating sample magnetometer (VSM, Riken Denshi, BHV-525) at 27 °C. The microwave absorption properties were measured using a vector network analyser (Agilent 8720ET) in the frequency range of 0.5–18.0 GHz. The Ni-10000 powder samples were pulverized and mixed with paraffin with a mass ratio of 3:1 and then pressed into a cylindrically shaped compact with an outer diameter of 7.0 mm and an inner diameter of 3.0 mm.

## Results and Discussion

### Morphology and Structure

**Fig. 2:**
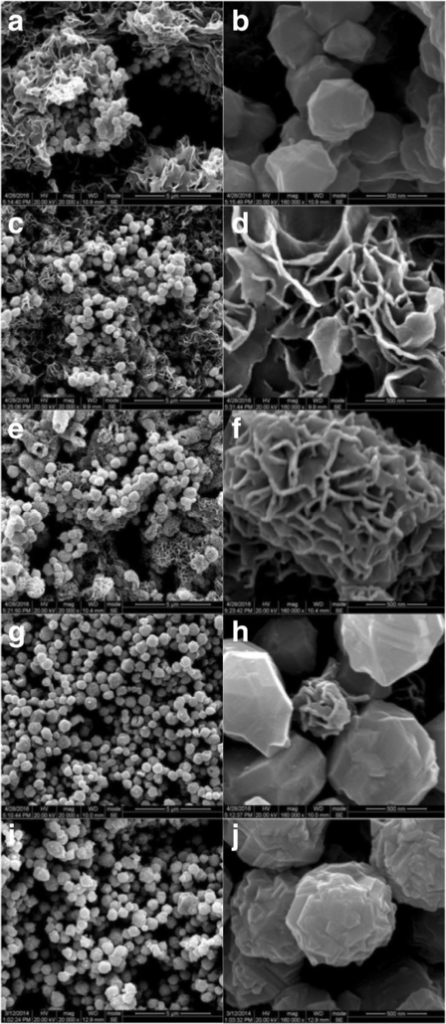
SEM images with two magnifications of Ni-0 (**a**, **b**), Ni-600 (**c**, **d**), Ni-2000 (**e**, **f**), Ni-6000 (**g**, **h**), and Ni-10000 (**i**, **j**) powders

To investigate the growth process of the Ni-SSs in detail, SEM was used to characterize the morphologies and structures of the Ni-0, Ni-600, Ni-2000, Ni-6000 and Ni-10000 powders. As shown in Fig. 2a, it was observed that the sheet-like hierarchical architectures (Ni-0 flower) were assembled by numerous interconnected flower-like petals with the thickness ca. 40 nm. Further observation from Fig. 2b showed that a few nanoparticles (Ni-0 sphere) were nearly spherical in shape with a large size range of approximately 200~500 nm. Fig. 2c, d clearly exhibited the Ni-600 (Ni-600 flower and sphere) morphology consisting of flowery structures with an average length of ~1000 nm and nanospheres with an average diameter of ~500 nm. All nanoplates in the flowery bundles seemed to grow from their centers. More interestingly, Fig. 2e, f depicted that the individual broken structures of Ni-2000 (Ni-2000 flower) with interior void between core and shell could be seen clearly. However, the sample was not highly uniform with different nanostructures such as flowers, spheres, and tubes. And the hollow core-shell structures had a large size range of 200~3000 nm. Close observation showed that these 3D flower-like hollow structures were made of dozens of crispate nanoflakes, which was similar to the Ni-600 flowers. Then, almost all the nanoparticles of the Ni-6000 powder possessed uniform sphere-like shape with an average diameter of 500 nm and only a few small flower-like nanospheres were found in Fig. 2g, h. It seemed to be the termination stage of the flower-like hierarchical architectures. There was no indication of any flower-like morphology in the Ni-10000 powder (Fig. 2i). All the samples were uniform nanospheres with a wide size range of approximately 200~800 nm. More detailed morphologies were displayed in Fig. 2j, showing that the submicron spheres were composed of massive nanoparticles with different shapes and sizes. This implied that almost all of the nanoparticles were self-assembled into sphere-like hierarchical aggregated particles. In addition, the surface of these polyhedral spherical particles was not smooth but rough with irregular polyhedron indicating that the polyhedral spherical particles might be built from smaller subunits with the random aggregation process.

**Fig. 3:**
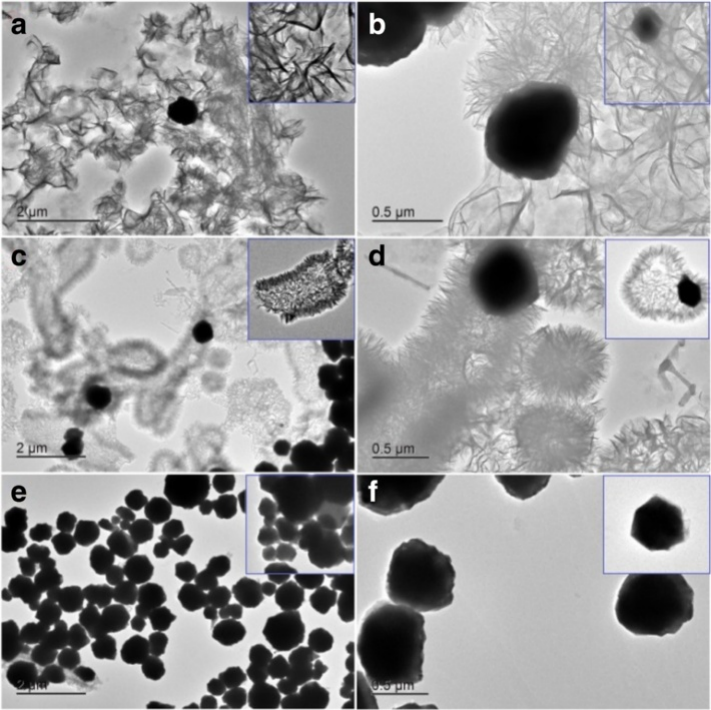
Typical TEM images with two magnifications of Ni-0 (**a**, **b**), Ni-2000 (**c**, **d**), and Ni-10000 (**e**, **f**) powders

The microstructure of the Ni-0, Ni-2000, and Ni-10000 powders was further investigated with TEM. As shown in Fig. 3a, b, the Ni-0 flower-like structures were randomly scattered with obvious agglomeration and consist of plentiful nanoplates, which was similar to the SEM observation. Besides a large number of flower-like microstructures, a minority of solid-core nanospheres also coexisted on the surface of the nanoplates. It was the fact that the nanospheres could be successfully synthesized in spite of the absence of any surfactant PEG. This was probably due to the tendency of nanoparticles to aggregate in aqueous state [33]. However, with the addition of medium length PEG-2000 polymer chain, most of the large flower-like hierarchical architectures disappeared and the well-defined hollow architectures were formed. Figure 3c, d shows that the pale canter together with the dark edge and the shell wall built from several tiny nanoplates was the evidence for the flower-like hollow submicron spheres. Simultaneously, many solid-core nanospheres also coexisted on the surface of the nanoplates. In addition, lots of micron-sized, multi-hollow hierarchical tubes could be prepared through this process. As shown in Fig. 3e, f, only pure metallic Ni nanospheres were obtained and randomly scattered with a diameter distribution of 200~800 nm. This was possibly because the PEG molecules with long length of polymer chain could control the agglomeration and growth of nanoparticles or nanosheets.

**Fig. 4:**
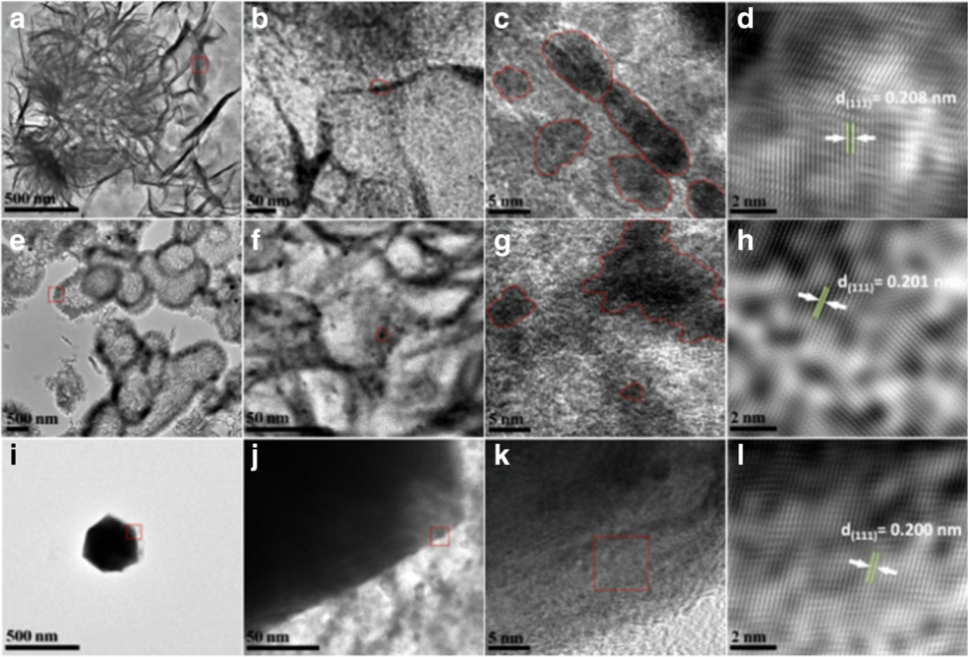
HRTEM images of Ni-0 (**a**–**d**), Ni-2000 (**e**–**h**), and Ni-10000 (**i**–**l**) powders with various magnifications

Figure 4 shows the high-resolution TEM (HRTEM) images of the Ni-0, Ni-2000, and Ni-10000 powders with various magnifications. The right row (Fig. 4d, h, l) shows the corresponding inverse fast Fourier transform (FFT) images. Figure 4a–c clearly shows that the nanopetal bundles of Ni-0 flower were made of numerous nanoparticles of ca. 8 nm in size. Similarly, a large amount of nanoparticles were homogeneously distributed among the flower-like hollow structures of Ni-2000 in Fig. 4e–g. The HRTEM images (Fig. 4i–k) further demonstrated that the pure Ni spherical particles of Ni-10000 had a sphere-like shape; a very small concentration gradient could be observed from the heart to the outer part of the particle, and Ni is slightly poorer in the outer being than the heart. It seemed that most of them were solid-core structures, and these particles tended to coalesce, probably due to the magnetic interactions which arisen from their crystal structure [34]. The overview of the HRTEM images showed the well-defined lattice planes with perfect crystallinity, which proved a very high degree of crystallinity. The three sets of lattice fringes could be observed clearly with spacing of 0.208 nm (Fig. 4d), 0.201 nm (Fig. 4h), and 0.200 nm (Fig. 4l). The spacing was close to the (111) interplanar distance of the face centered cubic Ni structure (0.203 nm) [35]. It was evidenced that the flower-like microstructures, hollow architectures, and Ni spherical particles were all built from small Ni nanoparticles. In addition, it was also found that point defects were present in the resultant microstructures, indicating that the Ni microstructures were of a polycrystalline structure.

**Fig. 5:**
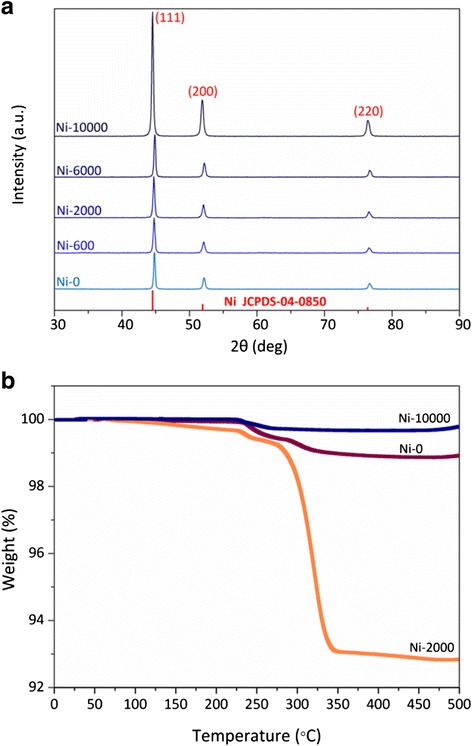
**a** XRD patterns and **b** TGA curves of Ni-0, Ni-2000, and Ni-10000 powders

In order to further confirm the structural features, the crystal information and phase purity of the as-synthesized Ni-0, Ni-600, Ni-2000, Ni-6000, and Ni-10000 powders were recorded by XRD. As shown in Fig. 5a, there were only three characteristic diffractions peaked at 44.5°, 51.9° and 76.4° in the XRD patterns. These peaks correspond to three indexed planes, (111), (200), and (220), respectively, which match well with the values of the FCC phase Ni reported in the database of JCPDS Card (No. 04-0850). It is obvious that the intensity of these three peaks increase with the increasing of the PEG molecules chain. Only the intensity of diffraction peaks of Ni-10000 powder increase significantly indicating that Ni-10000 has more homogeneous, well-defined architectures and better phase purity. Meanwhile, no other peaks were observed except the peaks of Ni, indicating that the samples with different architectures obtained by this method still consist of a pure phase [36, 37].

Then, thermal properties of the Ni-0, Ni-2000, and Ni-10000 powders were characterized using TGA under a nitrogen atmosphere. The TGA curves were shown in Fig. 5b. The total weight losses of the Ni-0, Ni-2000, and Ni-10000 powders at 500 °C were 1.1, 7.2, and 0.3 % respectively, showing excellent thermal stabilities and high purity. However, the Ni-0, Ni-2000, and Ni-10000 samples showed a two-stage decomposition process: the maximum-rate decomposition temperatures of the first stage were at 241.5, 235.8, and 231.4 °C, respectively; the maximum-rate decomposition temperatures of the second stage were observed at 303.6, 321.4, and 253.1 °C, respectively. The thermal results reveal that the first stage may be attributable to the thermal degradation of the organic components. Moreover, the second decomposition as well as increased weight losses corresponds to the decomposition reaction of the Ni which is probably related to the consequence of Ni structural crystallization [38].

**Fig. 6:**
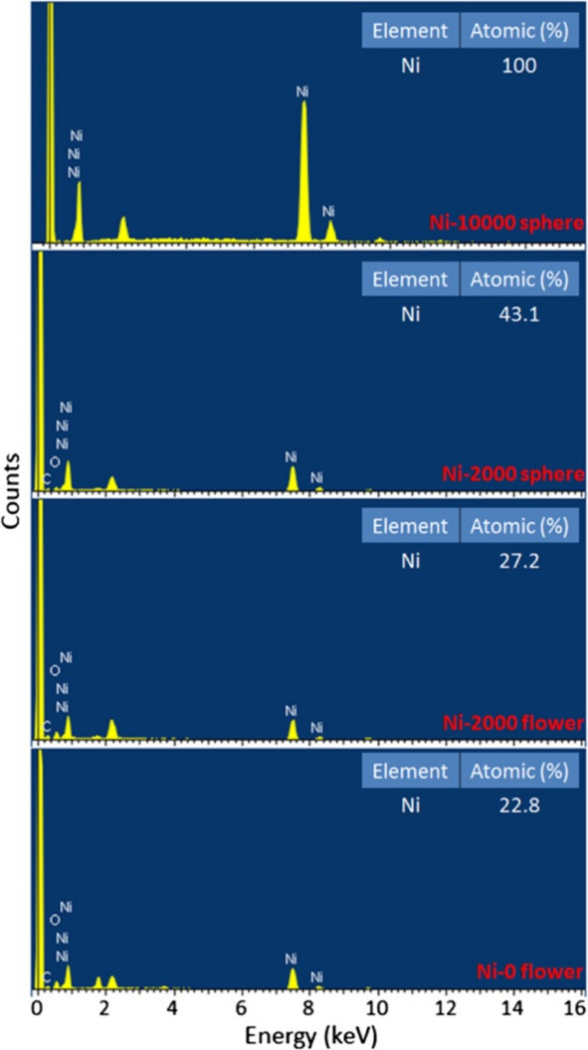
EDS spectra of Ni-0, Ni-2000, and Ni-10000 powders

Furthermore, the elemental composition of the Ni-0 (flower), Ni-2000 (flower and sphere), and Ni-10000 (sphere) powders by EDS in SEM is presented in Fig. 6. It is found that the Ni-0 and Ni-2000 is mainly composed of Ni, oxygen, and carbon. After the addition of PEG surfactant, the Ni content of Ni-2000 flower-like architectures slightly increased because of the aggregation of Ni nanospheres. Nevertheless, the Ni content of Ni sphere is significantly higher than that of Ni flower in the same sample (Ni-2000), showing the different formation mechanisms between Ni flowers and Ni spheres. The result also showed that surface of the flower-like nanoplates and hollow nanostructures may be partly oxidized or some surfactants were absorbed on the surface. More importantly, the Ni peak of Ni-10000 sphere was only observed and the element Ni content is 100 % with no other impurities. Combined with the abovementioned results, we conclude that the uniform pure Ni-SSs have been successfully synthesized through a simple solvent-thermal route with controllable surfactant PEG polymer chain lengths.

### Formation Process

**Fig. 7:**
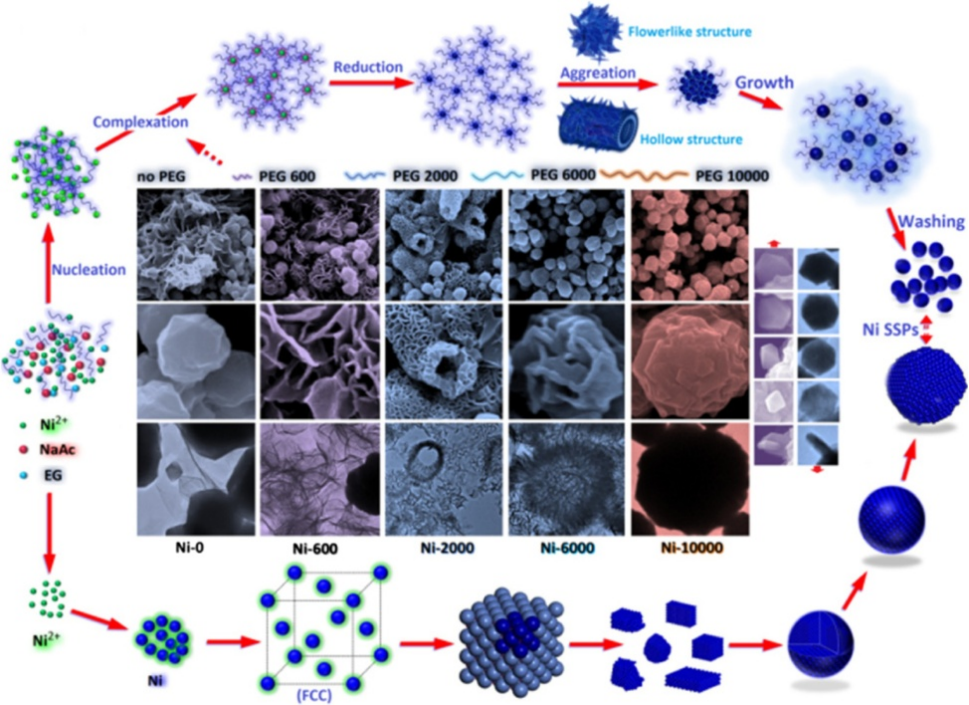
Schematic illustration of the morphological evolution process of the Ni submicron spheres

The solvothermal method as a controllable technique for the formation of microstructure involves the use of autoclaves or other high pressure vessels. Generally, the inorganic microstructures undergo fast nucleation and growth in an aqueous system with high temperature and pressure [23]. For our controllable surfactant PEG-assisted experiments, a stepwise growth mechanism is rationally proposed, and a schematic illustration of the morphological evolution process of the Ni submicron spheres is illustrated in Fig. 7. On the basis of SEM, TEM observations, and XRD results, the first possible formation route most likely begins with a reduction process, and the chemical reactions that resulted in the soluble Ni cation generation can be schematized as follows: [39]

1 $$ {\mathrm{H}\mathrm{O}\mathrm{CH}}_2{\mathrm{CH}}_2\mathrm{O}\mathrm{H}\to {\mathrm{CH}}_3\mathrm{C}\mathrm{H}\mathrm{O} + {\mathrm{H}}_2\mathrm{O} $$

2 $$ 2{\mathrm{CH}}_3\mathrm{C}\mathrm{H}\mathrm{O}\overset{{\mathrm{Ni}}^{2+}}{\to }{\mathrm{H}}_3{\mathrm{CCOCOCH}}_3 + {\mathrm{H}}_2\mathrm{O} + \mathrm{N}\mathrm{i}\ \downarrow $$

It can be assured that the Ni-0 flower-like nanoplates and nanospheres can be obtained without any surfactant and special capping ligands, and so it is reasonable that the Ni spheres were generated in the liquid polyols phase by a duplicative oxidation of acetaldehyde previously produced by dehydration of EG. Additionally, we may think that there are two other mechanisms that may be involved in the formation of three Ni hierarchical architectures including two basic mechanisms: the Ostwald ripening process and the aggregation growth process [40, 41]. At the beginning of the nucleation process, ethylene glycol as a high-boiling-point solvent [42, 43] and a reducing agent has been effective polyol reaction medium, which may act as a bridge to resolve and deliver the free Ni^2+^ ions [44] at an elevated reaction temperature (200 °C). According to the Ostwald ripening mechanism, the free Ni cations were nucleated in the EG solution. Afterward, it is likely that the Ni cations reacted with anhydrous sodium acetate to form a relative stable structure of the metal precursor, which is analogous to sodium formate in the literature [45]. Although anhydrous sodium acetate acts as an electrostatic stabilization agent to prevent particle agglomeration, it was found that no dark solid products were obtained in the absence of anhydrous sodium acetate. Our own experimental evidence has led us to believe that anhydrous sodium acetate plays an important role in the formation of metal precursors. In fact, in a generalized hydrothermal process, sodium borohydride or hydrazinium hydroxide as a complexing agent as well as a strong reducing agent has been often used for the metal salts to produce small metal particles both in aqueous and non-aqueous solutions [31, 37]. However, some complications can arise with the corresponding metal borides if the stoichiometry is not carefully adjusted. Therefore, in this work, the similar Ni^2+^ complexes can also be formed without the special complexing agent. Under appropriate solvothermal conditions, the similar Ni^2+^ complexes as the precursor could decompose and release free Ni^2+^ to form nanocrystalline Ni through the chemical reduction effect of EG. This might result from the fact that the probability of collision between Ni atoms and Ni nuclei has existed in the reduction reaction. In other words, once a few Ni nuclei were formed, the growth process would be superior to nucleation with enhanced reduction [31].

In the aggregation growth mechanism, Ni nuclei have a strong tendency to coalesce to form the initial aggregates with the interaction of PEG, serving as a surfactant and structure-directing agent and often altering the order of free energies on the metal surface [44]. According to the minimization of interfacial energy [46], smaller crystalline Ni nanoparticles may begin to aggregate together in the early stages and then lead to the generation of larger aggregates with the driving force for oriented aggregation. More importantly, to decrease the high surface energy, the nanoparticles preferentially agglomerate during their formation in the liquid-phase process [47]. Then, more uniform and larger nanostructures, such as flower-like nanoplates and hierarchical hollow structures, were gradually formed by tiny Ni nanoparticles with different PEG polymers. As the PEG molecules weight increases, the polymeric chain became longer and the entanglement became more pronounced. At the same time, hydrophilic groups in the PEG molecules adsorbed on the surface of Ni nanoparticles increased, leading to the decreasing of their aggregation; this also resulted in the disappearance of the flower-like hierarchical architectures. However, these Ni nuclei still further gathered the adjacent particles by magnetic dipole-dipole interaction and van der Waals force through self-organization in a common crystallographic orientation and joined these submicron particles at a planar interface [48, 49]. Consequently, the final polydisperse polyhedral Ni-SSs with a quasi-spherical shape and high purity can be obtained by the polyol reaction process when the long PEG-10000 polymer chains were added.

### Magnetic Properties

**Fig. 8:**
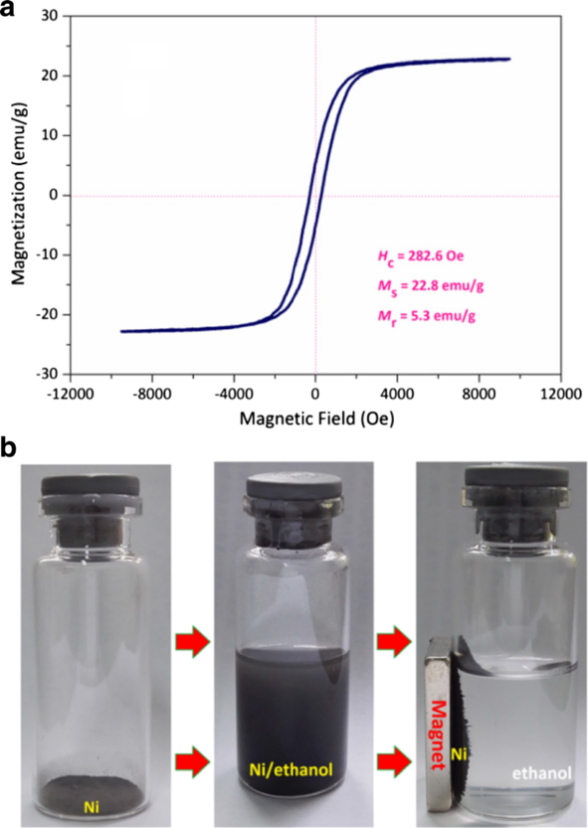
**a** Room-temperature magnetization curves and **b** photograph of Ni-10000 powder (*left*) dispersed in ethanol (*middle*) and their response to a magnet (*right*)

The Ni-based nanoparticles have potential for electromagnetic applications because their special nanostructures exhibited excellent microwave and electromagnetic absorption properties [50]. Magnetic properties were investigated and the hysteresis loops of the as-prepared Ni-10000 powder are shown in Fig. 8a, revealing the ferromagnetic behaviors. The remnant magnetization (*M*_r_) of the Ni-10000 powder is 5.3 emu g^−1^. The value of saturation magnetization (*M*_s_) is 22.8 emu g^−1^, which is much smaller than that of bulk fcc Ni (55 emu/g). Nevertheless, the coercive force value (*H*_c_) is about 282.6 Oe, which is relatively higher than that of bulk Ni (200 Oe) [51]. The large surface area of pure Ni-SSs induces high surface or shape anisotropy, leading to the increase of magnetic energy due to spin disorder. The increased magnetic energy is required to change the magnetic direction of dipoles resulting in the coercivity enhancement [52]. Many studies [21–23, 29–31, 34, 53–58] have reported Ni-based nanomaterials with different structures using different preparation methods, surfactants, reducing agents. Some of them have exhibited good performances in terms of their size and morphology control of the nanostructures, as shown in Table 1 [13, 17, 21, 29–31, 34, 35, 53–59]. However, the successful routes for fabrication of these nanomaterials have some disadvantages including high cost, poor stability, and complexity.

**Table 1:** The performance of Ni-based nanomaterials

Material	Preparation method	Structure	Surfactant	Reducing agent	H_c_ (Oe)	RL (dB)	Ref.
Ni	Solvothermal method	Nanocrystals	PVP	N_2_H_4_	167.0	–	[30]
Ni	Microwave irradiation	Icosahedral nanocrystals	TOPO	–	164.0	–	[53]
Ni	Thermal decomposition	Nanoparticles	ACA, TOPO	Alkylamine	290.0		[34]
Ni	Solution method	Nanoparticles	CTAB, TC_12_AB	N_2_H_4_	40.0	–	[31]
Ni	Solution method	Nanocones	–	N_2_H_4_	213.7	–	[54]
Ni	Solvothermal method	Flower-like nanostructures	–	PP	170.0	–	[55]
Ni	Solution method	Micro-flowers	EDA	N_2_H_4_	130.0	–	[56]
Ni	Solvothermal method	Hollow nanospheres	SDS	NaH_2_PO_2_	102.0	–	[57]
Ni	Solution method	Hollow submicro-spheres	–	NaH_2_PO_2_	–	−28.8	[29]
Ni	Solvothermal method	Submicro-spheres	PEG	–	282.6	−17.9	Our work
Ni	Electrodeposition	Branched nanowire	AAO	H_3_BO_4_	380.0	−19.0	[21]
Ni	Solvothermal method	Nanowire	–	Benzyl alcohol	186.2	–	[58]
Ni-B	Solution method	Nanoparticles	OA	KBH_4_	129.8	–	[59]
Ni-Pt	Solvothermal method	Hollow nanospheres	ET/EN	NaBH_4_	255	–	[35]
Ni-Cu	Electrochemical corrosion	Nanoparticle chains	AAO	H_3_BO_3_	–	–	[17]
Ni-NiO	Solvothermal method	Nanoparticles	TOPO, TOP	Oleylamine	–	–	[13]

In addition, to achieve the desired uniform architectures, the methods used are usually complicated, which always involve the use of various surfactants and reducing agents. In our work, a one-step synthesis method with no reducing agent and only one common surfactant has been applied. Thus, the pure Ni-SSs obtained by our solvothermal method exhibited relatively higher performances than most of other the nanostructures, and the shape of the as-obtained Ni nanostructures could be controlled by optimizing the only surfactant we used, thanks to one-pot synthesize route in the absence of any reducing agents. To examine the magnetic properties of the samples, the Ni-10000 powder was dispersed in ethanol easily at room temperature. But the mixture remains stable for only 15 min and then become clear. As shown in Fig. 7b, these pure Ni-SSs move quickly along the magnetic field as holding the sample close to a commercial magnet, indicating that it is possible to manipulate these magnetic Ni-SSs by an external magnetic field.

### Microwave Absorption Properties

**Fig. 9:**
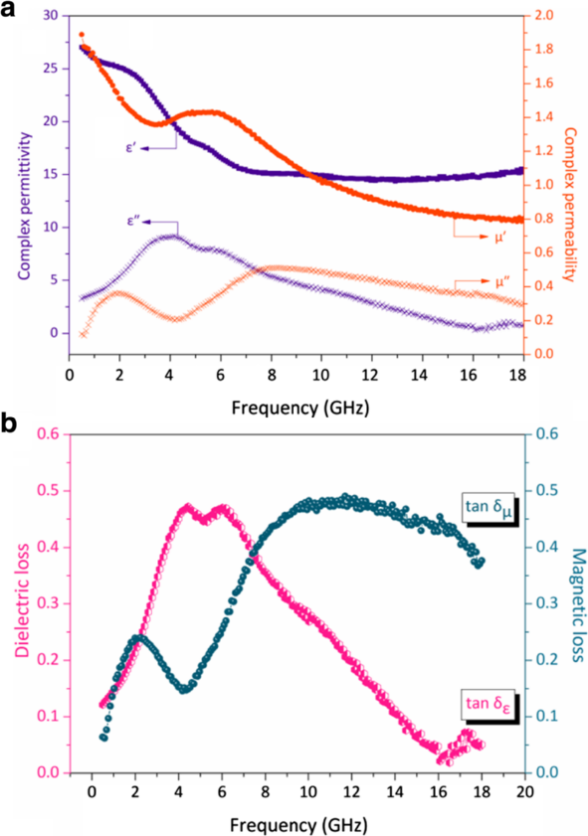
**a** complex permittivity and permeability and **b** dielectric loss and magnetic loss tangent of Ni-10000 powder as a function of frequency

The electromagnetic and microwave absorption behaviors of the Ni-10000 powder (pure Ni-SSs) were investigated in a frequency range of 0.5–18.0 GHz. Figure 9a shows the frequency dependence of the real and imaginary parts of the complex relative permittivity (*ε′*, *ε′′*) and permeability (*μ′*, *μ′′*) of the paraffin-based composites containing 75 wt% Ni-10000 powder dispersed in a paraffin matrix. Then, the electromagnetic properties can also be regard as the properties belonging to the Ni/paraffin composites. As shown in Fig. 9a, the *ε′* value of the Ni-SSs gradually decreases from 27.0 to 15.2 in the frequency range of 0.5–7.0 GHz. After that, the value of *ε′* has a negligible fluctuation in the range of 7.0–18.0 GHz. This is the fact that *ε′* is an expression of the polarizability of a material, which consists of dipolar polarization and electric polarization at microwave frequency [60]. In contrast with the *ε′* value, the *ε′′* demonstrates a great increase with one strong peak (9.2) at 4.1 GHz. However, the *ε′′* values decline over the range of 4.1–18.0 GHz. According to the free electron theory [61], *ε′′* = 1/(2*ε*_0_*πρf*), where *ρ* is the resistivity. The relatively low *ε′′* value of the material indicates a high electric resistivity. It is reasonable that dielectric loss is attributed to the lag of polarization between the interfaces as the frequency changes. The value of *μ′* has a rapid decrease from 1.9 to 1.3 with the frequency in 0.5–3.9 GHz and then has a minor peak value of 1.4 at 5.9 GHz, which comes from the cooperative effect of spin rotational resonance and domain-wall resonance of Ni nanoparticles [62, 63]. Moreover, the value of the *μ′′* has two peaks at 2.0 and 8.0 GHz in the whole frequency range, indicating the occurrence of strong wall resonance.

**Fig. 10:**
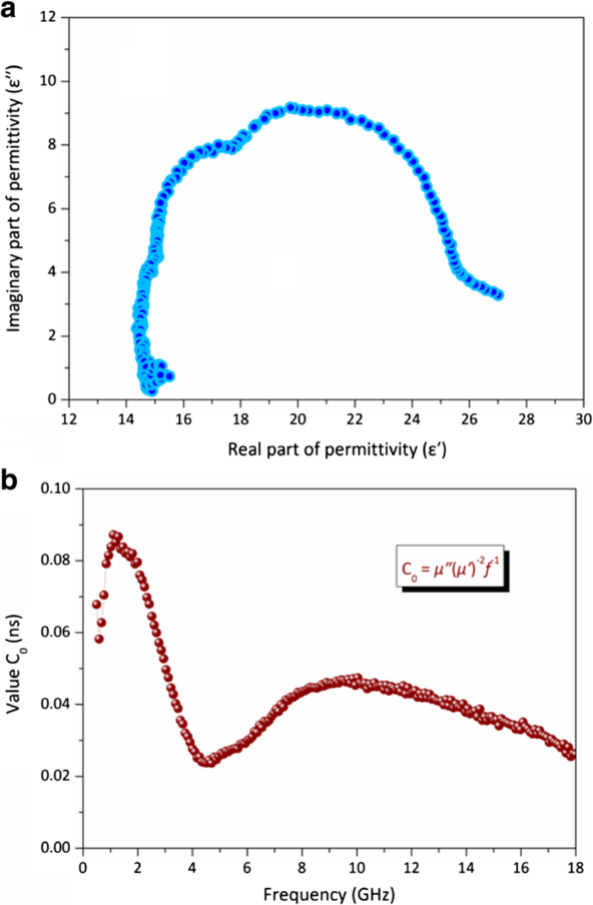
**a** dielectric Cole-Cole semicircle curve and **b** the value *C*_0_ of *μ*" (*μ*')^−2^ *f* ^-1^ versus frequency for Ni-10000 powder

As reported in Fig. 9b, the dielectric loss tangents (tan *δ*_ε_ = *ε′*/*ε′′*) exhibits two strong peaks around 4.4 and 6.0 GHz corresponding to the value of 0.47 and 0.47, respectively, indicating the occurrence of two strong resonances in the frequency range of 0.5–18.0 GHz. The observed results indicate that the Ni-10000 powder exhibits strong dielectric losses. It can be seen that the mechanisms contributing to the dielectric loss are associated with dominant dipolar polarization and relaxation phenomena [64, 65]. More importantly, the structures and shapes of a material have a profound effect on the absorption properties when an electromagnetic wave is radiated [66, 67]. Meanwhile, the magnetic loss tangents (tan *δ*_μ_ = (*μ′*/*μ′′*) also demonstrated similar characteristics of that of the *μ′′* with respect to frequency, showing two strong peaks in the 0.5–4.2 GHz and 4.2–18.0 GHz ranges, respectively. There are many reports concerning the factors making contributions to the magnetic loss mainly result from magnetic hysteresis, domain-wall displacement, and eddy current loss [68]. Then, it is worth noting that the Ni-SSs with addition dielectric interface, interfacial polarization, and more polarization charges on the rough surface of analogous spherical nanostructure could be interpreted as the results of electronic spin and charge polarization theory [69, 70], which make the behaviors of dielectric and magnetic losses more complex. Generally, it is noteworthy that the dielectric resonance is favorable to the improvement of microwave absorption properties. In order to identify the factors contributing to the permittivity property, the plotting of *ε′′* versus *ε′* is usually defined as the Cole-Cole semicircle (Kramers-Kronig Function), which displays a sole dielectric relaxation process. As shown in Fig. 10a, the permittivity may be consistent with one Debye dielectric relaxation model [71] and then the single Cole-Cole semicircle is clearly observed and distorted. This suggests that there are other mechanisms such as the motion of conducting electrons and the Ni-SSs/paraffin interfacial polarization [72], representing the contribution of the dielectric relaxation. Normally, the microwave magnetic loss of magnetic materials mainly results from magnetic hysteresis, eddy current loss, and domain-wall displacement [73]. It is worth noting that the magnetic hysteresis and domain-wall displacement usually appears in a very low frequency range (1–100 MHz) [74], and then the influences of the hysteresis and the domain-wall displacement can be ignored. If the magnetic loss from eddy current loss, the *C*_0_ (*C*_0_ = *μ"* (*μ'*)^-2^*f*^-1^ = 2*πμ*_0_*σd*^2^/3) values should be constants when the frequency varies, this is called the skin-effect criterion [75]. However, the value of *C*_0_ decreases drastically as a function of frequency in the range of 0.5–4.5 GHz (Fig. 10b). Afterward, the peak was observed at 10 GHz. These results indicate that the magnetic loss can be ascribed to the eddy current effect, which may be brought by the relatively larger particles agglomerated with small Ni nanocrystals. Therefore, the natural resonance mainly originates from the magnetocrystalline and shape anisotropies as well as eddy current loss, which can also contribute to the magnetic loss with the cooperation effect of the hysteresis and domain-wall displacement. Thus, it can be concluded that the main contribution to the microwave absorption results from the dielectric and magnetic loss, and the use of magnetic loss is more powerful.

It is well known that the microwave absorption ability of a material is generally evaluated by the reflection loss (RL). With the measured values of the complex permittivity and permeability, we then simulated the reflection loss of the prepared Ni-10000 powder based on a model for a single-layer plane-wave absorber using the transmission line theory [76]. In this model, the reflection loss usually can be evaluated by the following equation:

3 $$ \mathrm{R}\mathrm{L}\ \left(\mathrm{dB}\right) = -20\  \log \left|\frac{Z_{\mathrm{in}}-{Z}_0}{Z_{\mathrm{in}}+{Z}_0}\right| $$

where *Z*_0_ is the impedance of free space and is given by the formula:

4 $$ {Z}_0=\sqrt{\upmu_0/{\upvarepsilon}_0} $$

*Z*_in_ is the input characteristic impendence of the free/absorber space system, which can be obtained from the following expression:

5 $$ {\mathrm{Z}}_{\mathrm{in}}=\sqrt{\upmu_{\mathrm{r}}/{\upvarepsilon}_{\mathrm{r}}}\  \tanh\ \left[\mathrm{j}\left(2\uppi \mathrm{f}\mathrm{d}/\mathrm{c}\right)\ \sqrt{\left({\upmu}_{\mathrm{r}}{\upvarepsilon}_{\mathrm{r}}\right)}\right] $$

where *f* is the frequency of the microwave, *c* is the velocity of light in free space, and *d* is the thickness of the absorber, *ε*_r_ (*ε*_r_ = *ε′*-j*ε′′*) and *μ*_r_ (*μ*_r_ = *μ′*-j*μ′′*) are the measured relative complex permittivity and permeability, respectively. The sensitivity of the reflection loss minimum is dependent on the thickness of the sample, which also affects the position of the frequency as one of the crucial parameters. Therefore, to investigate the influence resulting from the thickness, the absorber thickness is termed as matching thickness with different thicknesses.

**Fig. 11:**
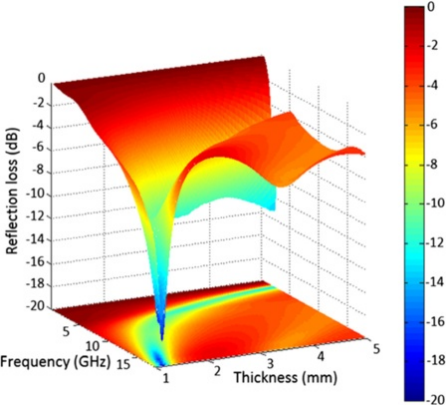
Three-dimensional representations of reflection losses of Ni-10000 powder

The three-dimensional representations of calculated RL of Ni-10000 powder are shown in Fig. 11. A minimum RL of −13.5 dB at the lower frequency band (0.5–5.0 GHz) was observed with the thickness of 5 mm. However, there is an increase of the RL peak intensity toward higher frequency with a decrease of the thickness. Furthermore, the minimal RL value is down to -17.9 dB at 17.8 GHz for the Ni-SSs as the thickness is 1.2 mm. It is obvious that the Ni-SSs present excellent microwave absorption properties in the whole frequency range. On the basis of the above mentioned mechanism, the Ni-SSs have intensities and positions of RL peaks corresponding well with mechanism of dielectric and magnetic loss. This result can be explained by the fact that the present Ni-SSs absorb the microwave mainly by dielectric loss at lower frequency. However, due to the high-magnetic nature, the microwave absorption is contributed to magnetic loss at a high frequency. It is important to note that the Ni-SSs with a self-aggregated structure have many irregular shapes and sizes, which result in intensive dielectric relaxation and complicated interface polarization [77]. More importantly, the microwave can be trapped for long periods in the uniform submicron structure system and the microwave energy is transformed to heat or other forms of energies, and finally dissipated. In fact, the minimal RL values of Ni-based nanomaterials corresponding to microwave absorption ability is strongly dependent on the structure, size and morphology, which are the key-factors for microwave absorption characteristic and intensity (Table 1). Compared to those of Ni-based nanomaterials, the pure Ni-SSs display excellent microwave absorption properties within multi-bandwidths disregarding the variation in lower frequency, which opens up opportunities for further development of microwave absorption applications.

## Conclusions

In conclusion, pure metallic nickel submicron spheres have been successfully synthesized by a facile and efficient one-step solvothermal method using different PEG agents. The as-synthesized Ni-SSs can be obtained without any further processing. The solid-cores were nearly spherical in shape with a wide diameter distribution of approximately 200~800 nm through the aggregation of small Ni nanocrystals. The pure Ni-SSs with a ferromagnetic behavior exhibited high coercivity values. Furthermore, the microwave absorption properties of the magnetic Ni-SSs researched were in the frequency range of 0.5–18.0 GHz. The minimum reflection loss reached −17.9 dB at 17.8 GHz with the thickness of 1.2 mm, suggesting excellent microwave absorption properties. Therefore, this one-pot synthesize route provides a universal and convenient way for preparations of larger scale pure Ni-SSs, which can be potentially used in industrial, commercial, and defense applications.
